# Trends in the Incorporation of Antiseptics into Natural Polymer-Based Nanofibrous Mats

**DOI:** 10.3390/polym16050664

**Published:** 2024-02-29

**Authors:** Lenka Piskláková, Kristýna Skuhrovcová, Tereza Bártová, Julie Seidelmannová, Štěpán Vondrovic, Vladimír Velebný

**Affiliations:** 1 Contipro a.s., Dolní Dobrouč 401, 561 02 Dolní Dobrouč, Czech Republic; 2 Nanotechnology Centre, Centre for Energy and Environmental Technologies, VŠB—Technical University of Ostrava, 17. Listopadu 2172/15, 708 00 Ostrava, Czech Republic; 3 Centre of Polymer Systems, Tomas Bata University in Zlín, Třída Tomáše Bati 5678, 760 01 Zlín, Czech Republic

**Keywords:** nanofibers, electrospinning, antiseptics, hyaluronic acid, chitosan, nanoparticles, wound healing, bandage

## Abstract

Nanofibrous materials represent a very promising form of advanced carrier systems that can be used industrially, especially in regenerative medicine as highly functional bandages, or advanced wound dressings. By incorporation of antimicrobial additives directly into the structure of the nanofiber carrier, the functionality of the layer is upgraded, depending on the final requirement—bactericidal, bacteriostatic, antiseptic, or a generally antimicrobial effect. Such highly functional nanofibrous layers can be prepared mostly by electrospinning technology from both synthetic and natural polymers. The presence of a natural polymer in the composition is very advantageous. Especially in medical applications where, due to the presence of the material close to the human body, the healing process is more efficient and without the occurrence of an unwanted inflammatory response. However, converting natural polymers into nanofibrous form, with a homogeneously distributed and stable additive, is a great challenge. Thus, a combination of natural and synthetic materials is often used. This review clearly summarizes the issue of the incorporation and effectiveness of different types of antimicrobial substances, such as nanoparticles, antibiotics, common antiseptics, or substances of natural origin, into electrospun nanofibrous layers made of mostly natural polymer materials. A section describing the problematic aspects of antimicrobial polymers is also included.

## 1. Introduction

Nanofibrous materials have experienced a significant evolution in the past decade, eliciting considerable interest from various research institutions and technology companies, where they are processed into final products. Primarily prepared from polymers, these materials hold significant potential, especially within the textile, healthcare, and food industries. These materials consist of fibers with a diameter smaller than 1 µm, which create a porous network in the form of nonwoven textile. The electrospinning method is prevalent in the production of nanofibrous layers. Two factors contribute to this fact: learning the principles of electrospinning is relatively simple, and the electrospinning process could be upgraded to allow mass production. Using this technology, it is possible to prepare homogeneous nanofibrous layers with sufficient weight, which could be further processed or modified for specific applications [1,2].

Nanofibers can be prepared from a wide range of polymeric materials, provided they satisfy certain requirements pivotal to creating fibers with a defect-free morphology. The parameters of polymer composition are chosen based on the intended characteristics of the polymer solution, including an appropriate molecular weight and the associated viscosity of the solution, high solubility in the solvent system (ideally resulting in the creation of a “true solution”), and a surface tension conducive to the formation of the Taylor cone and stability of the fiber-forming jet. Electrospinning is suitable for use with both natural and synthetic polymers, with synthetic polymers displaying generally better spinnability (the polymer chains are more homogeneous). Often, a combination of both polymer types is preferred, with the synthetic material acting as a “fiber-forming polymer” [3]. This synergistic approach frequently results in enhanced mechanical properties attributed to the synthetic component, while the natural component promotes biocompatibility, non-toxicity, and improves overall biodegradability or resorption capacity. The fiber-forming polymer can also protect natural materials that are labile in high-voltage environments.

The transformation of polymer materials into (nano)fibrous meshes is the solution of the future. Current research and development in the field of polymer materials focus mostly on easily degradable materials based on natural polymers, which are produced with technologies and processes that do not burden the environment and whose decomposition does not result in the formation of toxic degradation products. Another benefit of natural polymer materials is that they could inherently possess other beneficial properties as well, e.g., antimicrobial activity, antioxidant, and anti-inflammatory properties, and they can induce hemostasis and support wound healing, cell adhesion, and proliferation [4,5]. At the same time, the nanofibrous structure mimics the extracellular matrix, with the small pore size facilitating gas exchange while impeding the passage of bacteria and viruses. Thus, nanofibrous materials based on natural polymers exhibit significant potential, particularly in biomedical applications (tissue engineering and wound healing), and their properties may far surpass those of conventional materials [6].

Antimicrobial activity is an especially important property of nanofibrous materials applied in the medical and even food sectors, where these materials face environments that are highly conducive to the development of microbial growth and the contraction of unwanted infections. A nanofibrous structure that does not have an antimicrobial effect by itself can be modified by adding an antimicrobial agent. There are many ways of incorporating an antimicrobial agent into a nanofibrous network—the simplest one is to add an antimicrobial additive to a spinning polymer solution. The other, increasingly complex methods consist of spraying an antimicrobial additive solution onto an already spun fibrous network, adsorbing the additive to the surface of the nanofibers by soaking the layer in a solution containing the additive, and preparing structurally more complex core–shell fiber structures, in which the antimicrobial additive is part of either the core or the shell. The incorporation of antimicrobials and other additives, which can be partially degraded by high voltage, could be potentially problematic. However, their degradation can be prevented, for example, by using the core–shell technology or polymeric and non-polymeric nanoparticles as a secondary transport drug delivery system for such labile components. Core–shell technology produces fibers in which the additive is protected by a polymer shell. In the latter case, the antimicrobial additive is incorporated into the nanofibrous structure together with the nanoparticle.

Antimicrobial agents themselves can be of both organic and inorganic origin. Their nature does not affect their effectiveness. They can be divided into groups according to the microorganisms they primarily act against—antibiotics (bacteria), antifungals (fungi, yeast), antivirals, and antiparasitics. Based on the specific type of antimicrobial effect, antimicrobial agents can be divided into microbiostatic agents, which inhibit the growth of microorganisms and microbicidal agents that can kill the microbes [7]. The nomenclature further differentiates disinfectants, which kill microbes on non-living surfaces, antiseptics, which are applied to living tissue (e.g., healthcare personnel handwashes, surgical scrubs, and irrigation solutions), and antibiotics, which are usually taken orally as drugs, but could also be administered topically. In practice, a wide range of antimicrobial agents are used. As each group has its own mechanism of action, specific types are carefully selected. The choice is influenced, among other things, by the intended application of the product, depending on which the product may be required to have either a broad-spectrum effect, in which case antimicrobial substances that are effective against the widest possible spectrum of microorganisms are chosen (e.g., broad-spectrum antibiotics), or, on the contrary, a highly specific effect, in which case one reagent is preferred.

Over the last decade, antibiotic resistance (AR) has been on the rise and has been often discussed. In 2019, the number of deaths due to AR was estimated at 5 million [8]. Before 2019, AR was one of the top global medical priorities; however, the COVID-19 pandemic eliminated established approaches to limit the growth of AR to achieve immediate eradication of all microbial pathogens, with potentially serious implications of increased AR in the future [9]. For these reasons, as well, the need to search for new antimicrobials is still relevant and meaningful.

This review is focused on describing the incorporation of various antimicrobial agents, including nanoparticles, essential oils, or synthetic antimicrobial agents, into nanofibrous mats based on natural or antimicrobial polymers, which might overcome the problem of AR. Attention has been primarily paid to substances widely reported in the literature in the last years, thus encompassing the most used antimicrobial agents. The list might not be exhaustive, as some specialties may have been inadvertently omitted. For comparison, a section describing the incorporation of conventional antibiotics into nanofibrous layers for topical treatment is also included.

## 2. Inorganic Antimicrobial Agents

The most widely used inorganic antimicrobial agents are undoubtedly nanoparticles (NPs) [10,11,12], with silver nanoparticles (Ag NPs) being the most common [10,13,14], but there are also studies that report using reduced graphene oxide [15] or modified clay minerals [16]. To incorporate reduced graphene oxide or carriers based on clay minerals into nanofibrous mats, the additive is mixed into the electrospinning solution, which is then often sonicated prior to electrospinning. One of three approaches is usually used to incorporate inorganic nanoparticles: (1) by blending the prepared NPs into the spinning solution (blend)—NPs are dispersed by vigorous stirring [17] or ultrasound [18], (2) by adding a precursor or precursors into the spinning solution—NP synthesis prior to or after electrospinning [19], and (3) by using NP immobilization or in situ synthesis on the surface of the nanofibers during post-processing [11,20,21,22]. Naturally, each approach has certain advantages and disadvantages. Blend electrospinning is the easiest method for incorporating NPs into nanofibrous mats. As stated above, mechanical stirring or sonication are used to achieve homogeneous dispersion of NPs in the spinning solution; nevertheless, NPs can still agglomerate or aggregate due to van der Waals interactions. A change in the viscosity and electrospinnability of the polymer solution can occur, resulting in a lower quality of nanofibrous mats. Furthermore, antimicrobial efficiency can be reduced because NPs are anchored inside the polymer fibers and enveloped by the polymer. This reduces their bioavailability and NPs, or metal ions might not be released to interact with pathogenic microorganisms [19,23,24,25]. In situ preparation of NPs in a spinning solution produces better results in terms of NPs’ dispersion because components of the polymer solution can serve as stabilizing agents. However, some reagent residues may be toxic [23]. The spinning solution must be designed in such a way as to dissolve both the polymers and the NP precursor as well as produce smooth defect-free nanofibers when electrospun. Most procedures used for in situ synthesis of NPs on the surface of nanofibers during post-processing make use of the adsorption of precursors or metal ions onto nanofibers prepared from a spinning solution. This approach is, therefore, unsuitable for polymers that can dissolve or swell excessively in these solutions because the nanofibrous structure could be impaired.

### 2.1. Metal-Based Antimicrobial Agents

#### 2.1.1. Silver Nanoparticles

As previously stated, the most commonly used antibacterial NPs are silver nanoparticles (Ag NPs). Silver vessels as well as remedies containing silver were used for water and food storage and in the field of medicine (treatment of wounds and ulcers, dental hygiene, surgical sutures, etc.) since the time of the Persian Empire until almost the middle of the 20th century. The popularity of preparations containing silver declined as the use of antibiotics rose at the verge of the 20th century. However, due to the continued increase in antimicrobial resistance in bacteria, silver is returning to prominence in the field of antimicrobial applications [26,27]. It is generally known that materials in the form of nanoparticles often have different physical and chemical properties from those they display in their bulk form. Ag NPs possess strong antimicrobial properties, and at the same time, the risk of microbes developing resistance to their effects is low because the antimicrobial action they produce involves several mechanisms. Ag NPs have the ability to penetrate bacterial cell walls, change the structure of cell membranes, increase the permeability of cell membranes, produce reactive oxygen species (ROS), and interrupt the replication of deoxyribonucleic acid by releasing silver ions [28,29]. The toxicity of Ag NPs is still being discussed. Some studies claim that Ag NPs have low toxicity for human cells. Nevertheless, their toxicity depends on the manner of administration used, their size, shape, concentration, Ag^+^ ions release, etc. [30,31,32].

Studies dealing with nanofibers with Ag NPs are summarized in Table 1. The studies are divided by the way Ag NPs were incorporated into nanofibrous mats made from natural polymers or a combination of natural and synthetic polymers. The NPs that have been reported the most in these studies are nanofibers made from cellulose acetate, which was in many cases subsequently regenerated in the post-processing stage to cellulose by NaOH treatment [10,33,34,35]. Synthetic polymers include the commonly used polyvinylalcohol (PVA), polyvinylpyrrolidone (PVP), and polycaprolactone (PCL). Synthetic polymers were added into the spinning solution to enhance its spinnability [17,19] or the mechanical properties of the produced nanofibers [36]. 

Only a few of the studies report the use of blending for the incorporation of Ag NPs into nanofibrous mats. To incorporate Ag NPs into nanofibrous mats made from hyaluronic acid (HA) and PVA, El-Aassar et al. (2020) [17] first reduced Ag NPs using polygalacturonic acid (PGA), which also served as a stabilizing agent. Even though Ag NPs were dispersed in the spinning solution merely by mechanical stirring, they were distributed homogeneously in the produced nanofibers. The prepared nanofibrous mats produced a stronger antibacterial effect and induced faster wound closure compared both to the control and a commercially available ointment. Similarly, Fereydouni et al. (2023) [37] prepared the Ag NPs with chitosan (CHIT) as a stabilizing agent. For electrospinning, a solution of poly(ethylene oxide) (PEO) was added to the mixture. Resulting nanofibers had good antimicrobial activity against both Gram-positive and Gram-negative bacteria. To neutralize the possible toxicity of Ag NPs, Jatoi et al. (2019) [38] immobilized Ag NPs on the surface of TiO_2_ NPs using a polydopamine coating, which served simultaneously as an adhesive, reducing, and stabilizing agent. The Ag NPs/TiO_2_ NPs nanocomposites were incorporated into the spinning solution by stirring and sonication and were, therefore, well dispersed in the produced nanofibers. The prepared nanofibrous mats displayed antimicrobial activity against *Escherichia coli* and *Staphylococcus aureus* for up to 72 h.

Another method, which was reported by only a handful of the reviewed studies, is the incorporation of Ag NPs into nanofibers by reducing an Ag precursor to NPs in the spinning solution. Maharjan et al. (2017) [13] mixed a precursor (AgNO_3_) in a zein/polyurethane solution in dimethylformamide (DMF), which served as the reducing agent. They were able to control the size of the produced Ag NPs by adjusting reduction time—the longer the time, the bigger the NPs. Ag NPs of 5–30 nm in size were uniformly distributed on the surface of the fibers. Eghbalifam et al. (2020) [19] dissolved AgNO_3_ in an aqueous solution of gum Arabic (GA), which was then added to a PVA/DMF solution and left overnight. Both GA and DMF acted as reducing agents, and GA also acted as a stabilizer. The produced nanofibrous mats inhibited the growth of bacteria but did not hinder the proliferation of fibroblasts, which makes them a promising material for the treatment of the infected wounds. 

The most reported method of preparation of Ag NPs-coated biopolymer nanofibers is the reduction of silver ions to Ag NPs during post-processing. Nanofibrous mats that have been prepared in advance are immersed in an aqueous solution of silver nitrate (AgNO_3_) for various periods of time (30 min to 24 h), and the reducing agents and procedures differ in every study. The most frequently used reducing agent is an aqueous solution of sodium borohydrate (NaBH_4_). The NaBH_4_ solution can be added directly to the AgNO_3_ solution containing nanofibers [10] or nanofibrous mats can be immersed in the NaBH_4_ solution as a second step [25,35], with washing possibly included in between the two steps [14]. Moreover, silver ions can be reduced to Ag NPs by applying thermal treatment and using DMF as the reducing agent [33] or by using a glycol solution of sodium hydroxide [39]. Srivastava et al. (2019) [40] synthesized Ag NPs on the surface of tasar silk fibroin nanofibers via the “green route” using the leaf extract of *Tridax procumbens* as the reducing agent.

Some collectives decided to combine Ag NPs with other additives. Depending on the nature of the substance used, these combinations can produce a synergistic antibacterial effect, encourage wound healing, etc. For instance, Rather et al. (2023) [25] investigated the combination of rosemary essential oil and Ag NPs, observing a synergistic effect on antibacterial properties. Yang et al. (2020) [41] used side-by-side electrospinning and a special spinneret to simultaneously spin blends of PVP/ciproflaxin (CIP) and ethyl cellulose/Ag NPs and produce Janus nanofibers that could be used in wound healing. By using a combination of the two antibacterial agents, the authors created a material that has both a strong initial antibacterial effect caused by the immediate release of CIP and a long-term antibacterial effect produced by Ag NPs. Strong antibacterial and anti-inflammatory effects were also observed by Chen et al. (2022) [36], who prepared 3D nanofibrous sponges, decorated by a silver-metal organic framework (Ag-MOF) and curcumin. By incorporating dimethyloxallyl glycine into cellulose acetate nanofibers and synthesizing Ag NPs on their surface, the authors of [39] produced a nanofibrous material that had antibacterial properties and promoted wound healing. Sofi et al. (2021) [35] prepared a nanofibrous scaffold with incorporated particles of nano-hydroxyapatite that acted as nucleation sites inducing apatite growth. The mineralization provided the physiological environment necessary for the adhesion, growth, and proliferation of cells. To avoid microbial infections near the area of application of this scaffold, the surface was covered by Ag NPs. 

The presented studies claim that the prepared nanofibrous mats can be used in antibacterial applications in general or, more specifically, in the field of medicine as wound dressings, drug delivery vehicles, or for tissue engineering. However, only a few studies substantiated their claims by in vivo testing. In these studies, the prepared materials were tested on full-thickness wounds in pigs [14], rats [17], or on infected full-thickness wounds on mice [36]. The application of the new nanofibrous materials led to faster re-epithelization compared to commercially available dressings or creams. The 3D nanofibrous sponges made of gelatin/PCL with incorporated Ag-MOF and curcumin significantly promoted the healing of wounds infected with *S. aureus* without any adverse effects (redness, swelling, or suppuration), in comparison to the control. 

**Table 1:** Nanofibrous materials with incorporated Ag NPs.

Polymers	Additives	Incorporation	Antimicrobial Activity	Application	References
Polygalacturonic acid, hyaluronic acid, PVA	Ag NPs	Blend	*E. coli*, *S. aureus*, *B. subtilis*	Wound dressing	El-Aassar et al. (2020) [17]
Chitosan, PEO	Ag NPs	Blend	*E. coli*, *P. aeruginosa*, *S. aureus*, *S. mutans*	Wound healing	Fereydouni et al. (2023) [37]
Ethyl cellulose, PVP	Ag NPs, ciproflaxin	Blend	*E. coli*, *S. aureus*	Wound dressing	Yang et al. (2020) [41]
Cellulose acetate	Ag NPs anchored on TiO_2_ NPs	Blend	*E. coli*, *S. aureus*	Antibacterial applications	Jatoi et al. (2019) [38]
Polyurethane, zein	Ag NPs	Precursor reduced in the spinning solution	*E. coli*, *S. aureus*	Wound dressing	Maharjan et al. (2017) [13]
Gum Arabic, PVA, PCL (coating)	Ag NPs	Precursor reduced in the spinning solution	*S. aureus*, *E. coli*, *P. aeruginosa*, *C. albicans*	Wound dressing for infectious wounds	Eghbalifam et al. (2020) [19]
Cellulose acetate	Ag NPs	Post-process, in situ reduction	*E. coli*, *S. aureus*	Antibacterial applications	Kalwar et al. (2018) [10]
Cellulose acetate	Ag NPs	Post-process, in situ reduction	*E. coli*, *S. aureus*	Antibacterial applications	Jatoi et al. (2019) [33]
Cellulose acetate	Ag NPs	Post-process, in situ reduction	*E. coli*, *S. aureus*	Tissue engineering	Moon et al. (2021) [34]
Jellyfish biomass, PCL	Ag NPs	Post-process, in situ reduction	*E. coli*, *S. aureus*, *B. subtilis*	Wound healing	Nudelman et al. (2019) [14]
Tasar fibroin	Ag NPs	Post-process, in situ reduction	*E. coli*, *S. aureus*, *P. aeruginosa*, *S. epidermidis*	Drug delivery vehicle, tissue engineering	Srivastava et al. (2019) [40]
Cellulose acetate, polyurethane	Ag NPs, rosemary EO	Post-process, in situ reduction	*E. coli*, *S. aureus*	Wound dressing	Rather et al. (2023) [25]
Cellulose acetate	Ag NPs, hydroxyapatite	Post-process, in situ reduction	*E. coli*, *S. aureus*	Wound healing, bone tissue regeneration	Sofi et al. (2021) [35]
Cellulose acetate	Ag NPs, dimethyloxallyl glycine	Post-process, in situ reduction	*E. coli*, *B. subtilis*	Diabetic wound healing	Li et al. (2022) [39]
Gelatin, PCL	Ag-MOF, curcumin	Post-process, in situ reduction	*E. coli*, *S. aureus*	Wound dressing	Chen et al. (2022) [36]

#### 2.1.2. Zinc Oxide Nanoparticles

Zinc oxide nanoparticles (ZnO NPs) possess many attractive properties, which make them useful in many areas of human activity. Similar to silver, zinc oxide has been known for its antibacterial properties throughout history. Historical records show that ointments with ZnO were used to treat injuries and ulcers as early as 2000 BC. The exact mechanism behind the antimicrobial action of ZnO NPs is not yet known, but it is believed that ZnO NPs induce the production of excess reactive oxygen species, which can accumulate in the outer cell membrane or cytoplasm and release zinc ions. This would cause bacterial cell membrane disintegration, membrane protein damage, and genomic instability, resulting in the death of the affected bacterial cells. The antimicrobial activity of ZnO NPs is dependent on their size, shape, and photocatalytic activity. ZnO NPs have been approved by the FDA as a food additive, and they are generally recognized as safe and non-toxic in low concentrations. ZnO NPs can also absorb and block UV radiation and are, therefore, used as additives both in skin creams/sunscreens and textiles resistant to UV-VIS light [42,43,44]. 

Three methods of incorporation of ZnO NPs into nanofibrous mats have been reported, namely blend electrospinning, side-by-side electrospinning/electrospraying, and post-process immobilization of NPs on prepared fibers (Table 2). To disperse ZnO NPs in blend electrospinning solution, various authors used either stirring [12,45,46,47,48] or sonication [18,48,49], but little to no attention was paid to the homogeneity of NP distribution in the nanofibrous mats. Chen et al. (2019) [50] made use of the variability of the electrospinning process by using the side-by-side electrospinning/electrospraying process, in which a gelatin solution was spun and, simultaneously, a dispersion of ZnO NPs was sprayed. An image analysis of the produced nanofibers confirmed that NPs were present only on the surface of the nanofibers and were uniformly distributed. The method of post-process immobilization uses NPs that have been prepared in advance. Hasannasab et al. (2021) [20] first modified the surface of silk fibroin nanofibers by plasma treatment and acrylic acid grafting. In another study, a colloidal solution of ZnO NPs was applied to a nanofibrous mat that had been prepared earlier and left to dry [51]. In none of the procedures was the sample washed to remove non-adsorbed nanoparticles. However, a study focused on the release of Zn^+^ ions showed that Zn^+^ ions were gradually released over 5 days with no initial burst release. 

Some researchers also incorporated secondary additives, such as essential oils (EO) [18], antimicrobial polymers [12,45], antibiotics [48,49], and enzymes [20], into the nanofibrous mats they used for the preparation of antimicrobial dressings. Nanofibers with a combination of ZnO NPs and cinnamon EO displayed enhanced antibacterial effectiveness against *S. aureus* and cytocompatibility with human dermal fibroblasts compared to nanofibers loaded only with ZnO NPs. Most importantly, nanofibers loaded with a combination of additives proved to be more efficient than other nanofibrous solutions in healing full-thickness incision wounds infected with *S. aureus* in rats in terms of bacterial inhibition following a single application, healing speed, and the quality of skin structure recovery, which was verified by morphological, microbiological, and histopathological studies [18]. Ahmed et al. (2018) [12] and Ranjbar-Mohammadi et al. (2021) [45] chose two different methods of integration of CHIT, which is known for its antibacterial properties. The first collective of authors prepared nanofibers from CHIT and PVA, while the second collective incorporated a nanofibrillated CHIT/ZnO NPs composite into keratin/polylactic acid (PLA)/PVA nanofibers as an additive. In both cases, the combination of CHIT and ZnO NPs produced an excellent antibacterial effect. 

Most of the reported studies focused on preparing materials for wound healing, though some were intended for more specific applications, such as the treatment of burns or diabetic wounds. For diabetic wound healing, nanofibrous mats made from a combination of PVA/CHIT or PVA/CHIT/ZnO NPs were tested on incision wounds in diabetic rabbits and compared with untreated controls. Both types of nanofibrous mats accelerated wound closure, but wounds treated with the PVA/CHIT/ZnO NPs mats showed greater wound contraction and better quality of new tissue [12]. When used for the treatment of second-degree burns induced in mice and rats, silk fibroin/ZnO wound dressing materials decreased the inflammatory response, enhanced skin regeneration, and stimulated the growth of hair follicles, the formation of sebaceous glands, and the deposition of collagen to a greater degree than nanofibrous mats made solely from silk fibroin and untreated controls [20,46]. The positive effect of ZnO NPs on wound closure in vivo was also evident when the NPs were incorporated in CHIT/PVA nanofibers. Wound healing was accelerated, with almost complete wound healing achieved in 12 days. The wound space was matured, filled with granular tissue, and relatively thick squamous epithelium formed on the wound surface. Chitosan, which is degraded to N-acetylglucosamine during the wound healing period, supported fibroblast proliferation, collagen formation, and hyaluronic acid deposition at the wound site [47].

**Table 2:** Nanofibrous materials containing ZnO NPs.

Polymers	Additives	Incorporation	Antimicrobial Activity	Application	References
Silk fibroin, HA	ZnO NPs	Blend	*E. coli*, *S. aureus*	Burn wound healing	Hadisi et al. (2020) [46]
Chitosan, PVA	ZnO NPs	Blend	*E. coli*, *S. aureus*	Wound dressing, tissue engineering	Rezaei et al. (2023) [47]
HA, PVA, PEO	ZnO NPs, cinnamon EO	Blend	*S. aureus*	Wound dressing	El-Aassar et al. (2021) [18]
Chitosan, PVA	ZnO NPs, chitosan	Blend	*E. coli*, *S. aureus, P. aeruginosa*, *B. subtilis*	Diabetic wound healing	Ahmed et al. (2018) [12]
PLA, PVA, keratin	ZnO NPs, chitosan	Blend	*E. coli*, *S. aureus*	Tissue engineering applications	Ranjbar Mohammadi et al. (2021) [45]
PCL, gelatin	ZnO NPs, amoxicilin	Blend	*S. aureus*	Wound dressing	Jafari et al. (2020) [49]
Sodium alginate, PVA	ZnO NPs, ciprofloxacin	Blend	*E. coli*, *S. aureus*	Wound dressing	Sadeghi et al. (2023) [48]
Soy protein isolate, Eudragit	ZnO-halloysite nanotubes, allantoin	Blend	*E. coli*, *S. aureus*	Skin tissue-engineered constructs	Jaberifard et al. (2023) [52]
Gelatin	ZnO NPs	Side-by-side (spraying/spinning)	*E. coli*, *S. aureus*	Wound dressing	Chen et al. (2019) [50]
Silk fibroin	ZnO NPs (polydopamine coated), bromelain	Post-process immobilization	*E. coli*, *S. aureus*	Burn wound healing	Hasannasab et al. (2021) [20]
Gum arabic, PVA, PCL	ZnO NPs	Post-process immobilization	*E. coli*, *S. aureus*, *P. aeruginosa*, *C. albicans*	Wound dressing, antimicrobial and antibiofilm coating	Harandi et al. (2021) [51]

#### 2.1.3. Other Nanoparticles

There are several other types of metal-based nanoparticles but their incorporation into natural polymer-based nanofibrous mats is not commonly reported, such as in the case of Ag NPs and ZnO NPs. Thus, this section summarizes the literature describing the use of other metal-based nanoparticles, namely Fe_2_O_3_ NPs, TiO_2_ NPs, and Cu-based NPs, for antimicrobial applications in the medical field (Table 3). 

Similar to other presented types of NPs, the antimicrobial action of Cu-based NPs is attributed to the small size and high specific surface area. The mechanism involves release of copper ions and their interaction with bacterial cell walls. The ions are also able to adhere on and penetrate the cell wall, alter its permeability, or destroy it and cause leakage of cytoplasm. Moreover, the production of ROS, damaging DNA, denaturation of proteins, and altering the enzyme function, is involved [53,54,55]. Cu-based NPs can find applications in medicine (reducing hospital infections, treatment of wounds, and preventing colonization of medical devices) or for water purification. The advantage in comparison with widely used Ag and Au NPs is their lower price [53,54,55]. There are some examples of nanofibers/Cu-based NPs proposed for medicinal use found in the literature. Haider et al. (2021) [22] prepared cellulose nanofibers, and the CuO NPs were synthetized on their surface by reduction of the precursor. Nanofibrous mats showed good antibacterial efficacy against both G− and G+ bacteria, cytocompatibility, antioxidant activity, and the moisture vapor transmission rate, thus being a good candidate for wound healing applications. Lu et al. (2022) [21] developed chitosan nanofibers with CuS NPs and fucoidan immobilized on their surface. The antibacterial action is unique to other reported studies—the bacteria is killed by photothermal and photocatalytic bactericidal effects. CuS can convert near-infrared light into heat, and thus it produces local hyperthermia for thermal ablation of bacteria. The release of fucoidan and Cu ions promoted alkaline phosphatase activity of osteoblast cells and capillary tube formation of endothelial cells, which is suitable for treating wound or bone infections and for tissue regeneration, after further in vivo study. Elevated concentrations of Cu ions increase the risk of toxicity because they can interfere with the homeostasis of other metals, damage DNA, and generate reactive oxygen species. To avoid these possible toxic side effects of Cu ions caused by their high concentration, Zirak Hassan Kiadeh et al. (2021) [56] incorporated Cu-based MOF with gradual release of Cu ions into pectin/PEO nanofibers. In addition, folic acid for stabilizing the Cu-MOF and enhancing angiogenesis, fibroblast migration, and proliferation was incorporated. The prepared nanofibers exhibited antibacterial activity while being biocompatible, indicating that the Cu ions’ release was in the safe range.

The use for medicine (wound healing) applications was also described for Fe_2_O_3_ NPs. The nanoparticles were prepared by green synthesis using bacteria *Lactobacillus acidophilus* and *Lactobacillus plantarum* and then coated onto the gum Arabic/PVA/PCL nanofibers. The Fe_2_O_3_ NPs can penetrate the cell wall and cause cell membrane injury and cell death due to the small size and high specific surface area. Probiotic *Lactobacillus* bacteria can, on top of that, produce inhibitory compounds and reactive oxygen species; thus, these two agents act synergistically. The nanofibrous mats were able to inhibit bacteria, fungi, as well as biofilm by adsorbing the microorganisms onto the nanofibers while maintaining excellent cell viability [57]. 

Titanium dioxide nanoparticles (TiO_2_ NPs) are among the most used nanoparticles in different industrial and consumer products due to their catalytic and antimicrobial properties. TiO_2_ NPs are also used in many areas—building engineering (windows, tiles, wall paint, and coatings), agriculture (fertilizers and pesticides), environmental protection (water and sewage treatment), pharmaceuticals, food products, and the cosmetics industry [58,59]. The antimicrobial activity is related to several mechanisms—interaction between NP and bacterial surface, release of ions, and production of ROS under UV light [60]. Blantocas et al. (2018) [11] prepared chitosan nanofibers consequently coated by TiO_2_ NPs via the plasma-enhanced chemical vapor process for wastewater remediation. The original idea was to blend photo-catalytic metal oxide and chitosan with antimicrobial properties. However, pure chitosan nanofibers showed no inhibitory effect. On the other hand, composite material showed clear inhibition zones in disc diffusion assay, with their size depending on the plasma deposition time.

In a study by Shahverdi et al. (2023) [61], the use of cerium aluminate NPs (CeAlO_3_ NPs) in potential nanofibrous wound dressing was reported for the first time. NPs were dispersed in a solution of chitosan and PVA, together with cephalexin, an antibiotic model. Composite nanofiber mats with PCL were prepared by side-by-side electrospinning. The incorporation of NPs caused an increase in tensile strength and modulus. CeAlO_3_-incorporated nanofibers did not affect nanofiber biocompatibility, and fibroblast cells could better grow, differentiate, and cover the scaffold surface compared to the neat fibers. The prepared nanofibers could be a promising wound dressing material prepared by electrospinning directly onto a fabric dressing.

**Table 3:** Summary of various inorganic NPs incorporated in natural polymer-based nanofibrous mats.

Polymers	Additives	Incorporation	Antimicrobial Actitivy	Application	References
Pectin, PEO	Cu MOF, folic acid	Blend	*E. coli*, *S. aureus*	Drug delivery system	Zirak Hassan Kiadeh et al. (2019) [56]
Cellulose	CuO NPs	Post-process in situ synthesis	*E. coli*, *S. aureus*	Wound dressing	Haider et al. (2021) [22]
Chitosan	CuS NPs, fucoidan	Post-process immobilization	*E. coli*, *S. aureus*	Treating bone and wound infections, tissue regeneration	LuLu et al. (2022) [21]
Gum arabic, PVA; PCL coated	Fe_2_O_3_ NPs	Post-process adsorption	*E. coli*, *S. aureus*, *P. aeruginosa*, *C. albicans*	Wound healing applications	Harandi et al. (2022) [57]
Chitosan	TiO_2_ NPs	Post-process	*S. aureus*	Wastewater remediation	Blantocas et al. (2018) [11]
Chitosan, PVA, PCL	CeAlO_3_ NPs, cephalexin	Blend	*E. coli*, *S. aureus*	Wound dressing	Shahverdi et al. (2022) [61]

### 2.2. Non-Metallic Inorganic Antimicrobial Agents

This section presents other inorganic nanomaterials, including graphene derivatives and clay minerals, which have been used in recent years as fillers in nanofibrous mats based on natural polymers intended for antimicrobial applications (summarized in Table 4). Graphene derivatives should possess inherent antimicrobial properties; in the case of clay minerals, these only serve as carriers for other antimicrobial substances. 

Graphene and its derivatives (graphene-based materials—GM) have great mechanical and conducting properties, and they are usually used for preparation of nanofibrous nanocomposites with potential in tissue engineering or sensor technologies [62,63]. The reported antimicrobial activity of GM is attributed to the physicochemical interaction between GM and bacteria and physical damage of the bacterial membranes, leading to the inactivation of bacteria due do leakage of the intracellular matrix. The antimicrobial effect further includes various mechanisms, such as inducing oxidative and membrane stress, deterioration of cellular components (protein, lipids, and nucleic acids), or interaction with RNA/DNA hydrogen groups [64,65]. In recent years, graphene oxide or reduced graphene oxide was incorporated into silk fibroin nanofibers by blending the additive in the spinning solution. This method should suppress possible toxic effects of the nanofillers. The prepared nanofibrous mats were found effective against both Gram-positive and Gram-negative bacteria and biocompatible (in vitro) [15,66,67]. 

Inorganic nanoparticles based on clay minerals, such as halloysite nanotubes (halloysite NTs), montmorillonite, or kaolinite, are often used as carriers for drugs or nanoparticles. Clay minerals are generally chemically inert with good biocompatibility and low toxicity. Drug loading is influenced by many factors, including the surface area, cation exchange capacity, charge density, and swelling degree of clay [68]. Fatahi et al. (2021) [69] loaded levofloxacin, an antibiotic drug, into halloysite NTs, which were incorporated into sodium alginate/PEO by blend electrospinning. Resulting nanofibrous mats possessed biocompatible and good antimicrobial efficiency, which was slightly lowered in comparison to sodium alginate/PEO/levofloxacin nanofibers. Most importantly, in vitro release analysis indicated that nanocomposite fibrous mats have great potential for the sustained release of the antibiotic. Zou et al. (2020) [70] prepared chitosan/PCL nanofibers blended with chlorogenic acid/halloysite NTs. Chlorogenic acid is a naturally occurring plant-derived bioactive compound with antimicrobial and antioxidant properties. The composite nanofibrous mats showed improved hydrophilicity and long-term sustained release of chlorogenic acid driven by Fickian diffusion. However, the material was meant for its usage in the food industry, so no in vivo tests were performed.

Hydroxyapatite (HAp) is a bioceramic material with similar mineral components and crystal structure as mammalian bones. HAp is biocompatible, bioactive, osteoconductive, and stable (both chemically and thermally), but it lacks other certain important properties, such as mechanical strength or antimicrobial activity. However, HAp can be modified by incorporation of a wide range of ions into the crystal lattice while the crystal remains stable, introducing additional properties. The doping by naturally occurring elements (Mg, Zn, Mn, Cu, Sr, Ag, Ce, and Cu) with intrinsic antimicrobial activity could be used to turn HAp into a drug carrier, preventing bacterial infections and promoting other biological processes in tissue engineering [16,71]. Antibacterial activity against *S. aureus* and *E. coli* grew simultaneously with higher doping by Cu ions. At the same time, human fibroblast cells were cultivated on the scaffolds in vitro and were adhered to, spread, and proliferated both on fibers’ surface and inside the scaffold [55]. Similar results were reported by Kandasamy et al. (2020) [16], when the zone of microorganism inhibition increased with the increase in concentration of Zn and Mn co-dopants. Carboxymethyl cellulose/PVP/HAp were also found hemocompatible, non-toxic (supported the attachment and proliferation of HOS cells), and performed biomineralization activity in vitro. The material was proposed for bone tissue engineering applications after further in vivo studies. 

Ramírez-Agudelo et al. (2018) [72] incorporated HAp nanoparticles and doxycycline into gelatin/PCL nanofibrous mats for cancer therapy. Doxycycline is a well-known antibiotic with activity against cancer cells, and it was reported that even HAp nanoparticles themselves inhibit the proliferation of several kinds of cancer cells while supporting the proliferation of normal bone cells. The synergic dual-effect was confirmed by a higher cytotoxic effect on selected cancer cells and higher bacteria inhibition performed by combinational nanofibrous mats compared to single-agent nanofibers. Moreover, HAp nanoparticles might be used as drug carriers, similarly to nano-sized clay minerals. Wang et al. (2021) [73] adsorbed tetracycline hydrochloride (TCH) on the surface of HAp nanoparticles and the composite was then blended with the spinning solution and electrospun. The release of TCH from TCH/HAp/gelatin/chitosan nanofibers was significantly prolonged in comparison to the release of TCH from TCH/gelatin/chitosan nanofibers, from 4 to 14 days. The antibacterial activity was tested by the disc-diffusion method also on “pre-released” samples, meaning the samples were left in a release medium for certain time and then the antibacterial tests were conducted. TCH/gelatin/chitosan nanofibers showed prominent inhibitory zones after 1 and 4 days, and a small inhibition zone after 6 days of pre-release. In contrast, TCH/HAp/gelatin/chitosan nanofibers showed a distinctive inhibition zone even after 14 days of pre-release. The authors propose the prepared material as an attractive candidate for applications in wound dressings and drug release systems.

**Table 4:** Summary of other inorganic materials incorporated in natural polymer-based nanofibrous mats.

Polymers	Additives	Incorporation	Antimicrobial Activity	Application	References
Silk fibroin	Graphene oxide	Blend	*E. coli*, *S. aureus*	Wound dressing	Wang et al. (2018) [74]
Silk fibroin	Reduced graphene oxide	Blend	*E. coli*, *S. aureus*	Tissue engineering	Zhang et al. (2021) [15]
Silk fibroin	Reduced graphene oxide/TiO_2_	Blend	*S. aureus*	Tissue engineering	Zhang et al. (2021) [66]
Sodium alginate, PEO	Halloysite NT, levofloxacin	Blend	*E. coli*, *S. aureus*	Drug carrier, wound dressing	Fatahi et al. (2021) [69]
Cellulose acetate	HAp + Cu ions	Blend	*E. coli*, *S. aureus*	Regeneration of skin tissues	Elsayed et al. (2020) [71]
Carboxymethyl cellulose, PVP	HAp, doped by Zn and Mn	Blend	*E. coli*, *S. aureus*, *C. albicans*	Bone tissue engineering	Kandasamy et al. (2020) [16]
Gelatin, PCL	HAp, doxycyclin	Blend	*S. aureus*, *P. gingivalis*	Drug delivery	Ramírez-Agudelo et al. (2018) [72]
Gelatin, chitosan	HAp, tetracycline hydrochloride	Blend	*E. coli*, *S. aureus*	Antimicrobial wound dressing	Wang et al. (2021) [73]

## 3. Organic Antimicrobial Agents

In this section, attention is focused on organic antimicrobial agents. Among the most relevant groups are antimicrobial agents of natural origin, with various plant extracts involved, antibiotics, and synthetic antiseptics not acquired from natural origin. These categories of organic antiseptics were compared mutually and with the aforementioned inorganic antiseptics. The incorporation of these agents into nanofibers poses a significant challenge, mainly due to their hydrophobic nature. Consequently, the resultant material may not exhibit a homogeneous distribution of the antiseptic across its structure. Therefore, an evaluation of their efficacy against undesirable microorganisms was also conducted.

In agreement with the current trends of giving value to natural and renewable resources, the use of natural antimicrobial compounds, particularly in food and biomedical applications, becomes very frequent. To inhibit growth of undesirable microorganisms in food, or prevent the occurrence of infection, the antimicrobials can be directly added right into the product formulation, coated on its surface, or incorporated into the structure of the packaging/dressing material [75]. Additionally, agents with natural origin are more likely to be tolerated by human/organic tissue. However, this does not make them less effective compared to the above-mentioned inorganic antimicrobials. On the contrary, natural products are very often the original allergens, so it is necessary to use them in appropriate quantities [76]. Plants (e.g., herbs and spices, fruits and vegetables, seeds, and leaves) are the main source of natural antimicrobials and contain many essential oils that have a preserving effect against different microorganisms. Mainly, herbs and spices contain the most essential oils, and examples include rosemary, sage, basil, oregano, thyme, cardamom, and clove [77]. Organic agents in general are not only based on plant extracts but can also be synthesized—in a lab, or by microorganisms. This group of synthetic compounds includes the most common antiseptics and antibiotics, primarily used in medicinal practice. 

Antimicrobial activity, not only from a biomedical perspective, is related to compounds or elements that locally kill microorganisms or reduce their growth, without being toxic to surrounding tissues or surfaces [78]. Previous research has revealed the effectiveness of common organic antiseptics, such as chlorhexidine (CHX), triclosan, polyethyleneimines (PEI), etc., against different bacteria [79]. However, electrospinning of these compositions could be a challenge, as a wide range of material interactions between the free chains of natural polymers forming the nanofibrous matrix and antiseptics is possible. The choice of appropriate process and solution parameters is, therefore, key in order to maintain the efficiency of such a highly functional fibrous layer. Coaxial spinning technology, where the additive is protected by a polymer shell, is often chosen due to the protection of the incorporated substance [80]. The use of organic materials as antiseptics, in combination with natural polymers as a nanofibrous matrix, may also help increase the potential for successful biodegradability of such a material, while in contact with living tissue. 

### 3.1. Natural Antimicrobial Agents

As mentioned, the most numerous groups of organic antimicrobial agents are various plant extracts. Such substances are often obtained from tropical plants and contain various alkaloids or oils with a strongly lipophilic structure. Since these substances are poorly soluble in polar solvents, it is beneficial to use the formulation of an oil-in-water emulsion or coaxial spinning for their stable processing and effective incorporation into the fibrous network.

Natural antiseptic substances are often combined with a fibrous chitosan matrix, due to its inherent antiseptic function. In the study by Castellano et al. (2023) [81], the research highlights the promising role of chitosan (CHIT) and collagen (COL) electrospun nanofiber patches loaded with curcumin as advanced wound dressings with improved wound-healing and tissue-regenerating properties. Polyethylene oxide (PEO) was used as a fiber-forming promoter. To enhance the mechanical properties of the nanofiber matrix and its stability in a physiological environment, a crosslinking step was included by exposure to ammonia vapors or UV light, an approach that allows adjusting comparable properties of natural polymers with synthetic polymers. This step also affected the material’s porosity and gas permeability, which would typically disappear after soaking in water. 

Cumulative release and kinetic profile analysis of curcumin were monitored in PBS (pH 7.4, T = 37 °C). Three types of samples were compared—COL + curcumin, CHIT + curcumin, and composite COL + CHIT + curcumin for 72 h. In non-crosslinked samples, the highest amount of curcumin was released from the COL + curcumin matrix due to collagen’s willingness to swell in the aqueous environment. Conversely, in crosslinked samples, the highest amount of curcumin (almost 100%) was released from the composite COL + CHIT + curcumin, due to the reinforcement of the fibrous structure. The release rate stabilized after 5–10 h of the experiment, followed by prolonged release. Antimicrobial efficacy was tested on bacterial strains *E. coli*, *Pseudomonas aeruginosa*, and *S. aureus*. The COL + CHIT + curcumin composite demonstrated the highest resistance to bacterial growth, with the highest efficacy observed against *E. coli*. The composite chitosan–collagen membrane with embedded curcumin was also evaluated as the best substrate for cell adhesion for both fibroblasts and keratinocytes. A similar increased antibacterial effect of samples due to the addition of curcumin was observed in gelatin- and hydroxyapatite-based nanofiber layers [82]. The inhibitory zone was comparable for *E. coli* and *S. aureus*. For the comprehensive characterization of the material concerning targeted wound healing applications, it would be beneficial for both author groups to conduct future studies comparing different amounts of curcumin and their impact on maintaining antimicrobial effectiveness.

Other authors have also included chitosan, as a representative of natural polymers, with natural antimicrobial additives in their experiments. Chitosan was often spun in combination with synthetic hydrophobic PCL or PVP to improve the spinnability and mechanical stability of the nanofiber layers in a moist environment [83,84,85], or hydrophilic PEO or PVA. Hydrophilic fiber matrices are crosslinked before application, or the composition is adjusted to prevent fast dissolution but to allow gradual swelling [86,87]. Antimicrobial natural additives of various kinds are usually directly incorporated into the spinning solution, resulting in the formation of nanofibers with the additive both within and on the fiber surface. In the case of the PCL + CHIT + Propolis combination [83], nanofibers were prepared from a uniform solvent system. The ability of propolis to penetrate the nanofiber matrix was verified through release tests. Approximately 70% of the total additive amount was gradually released over 25 days. The safety and performance of nanofibers was verified in an in vivo study on male rats with second-degree burns, where nanofibers were used as a substitute for skin grafts. Wounds were analyzed after 3, 7, 10, and 14 days. A visible difference in the wound healing rate was observed on the 7th day after applying the sample to the wound. The presence of Propolis enhanced the tissue granulation process, re-epithelialization, and reduced the overall diameter of the defect. No development of infection was observed at the defect site. Similar results were achieved in the study by Kassem et al. (2023) [85], where Manuka honey was used as an antimicrobial agent and PVP + CHIT as the matrix. The preparation of the polymer solution was more demanding due to the specific viscosity of Manuka honey and the presence of TiO_2_ nanotubes, but the authors managed to prepare nanofibers with a smooth surface, which were finally crosslinked with glutaraldehyde vapors to improve the mechanical properties and vapor permeability. The healing potential was tested in vivo on male mice. The wound site was inspected after 1, 2, 4, 6, 8, 10, and 12 days. Accelerated healing was observed after 4 days of application, compared to commercially available bandages. Granulation in the wound was supported, leading to faster closure without apparent damage, scarring of surrounding tissue, or the occurrence of an inflammatory reaction. The wound was completely healed after twelve days.

Other natural fiber-forming polymers suitable for combining with natural antiseptics are hyaluronic acid or cellulose. Authors Snetkov et al. (2020) [88] and Ionescu et al. (2021) [89] utilized the biocompatibility of hyaluronan and created nanofiber layers with antimicrobial effects. Hyaluronan was combined with PEO or a specific solvent system (dimethyl sulfoxide—DMSO) to promote the spinnability. In the case of the HA and PEO combination [89], where a wide spectrum of substances was used as additives—e.g., L-arginine, Propolis, α-bromo-naphthalene, *Calendula officinalis*, insulin, or Manuka honey—nanofibers with defect-free morphology and a comparable fiber diameter for all fiber combinations were prepared. This shows that additives are homogeneously dispersed in the fiber structure. The antimicrobial function of nanofibers was tested on microbial strains *E. coli*, *P. aeruginosa*, *S. aureus*, and *Candida albicans*, commonly found in infected open wound environments. Combinations of HA + PEO with the additives Propolis + *C. officinalis* and Manuka honey + L-arginine exhibited the highest average inhibitory zones, specifically against *S. aureus*, but not against *C. albicans*. None of the tested samples, however, showed higher effectiveness than ciprofloxacin, used as a control. The authors should consider increasing the concentration of additives for enhancing the antimicrobial effect of samples in their future experiments. Nevertheless, Ionescu et al. (2021) conducted a similar study with chitosan [90], in combination with PEO and the mentioned additives, and here, a significantly higher antimicrobial efficacy—specifically for the CHIT + PEO combination with Propolis + *C. officinalis*—was recorded, including against *C. albicans*. The presence of an antimicrobial natural polymer thus plays a significant role in antimicrobial activity. In case of hyaluronan spun from DMSO, with the addition of curcumin and usnic acid [88], non-homogeneous nanofiber samples with low areal weight were obtained, due to the instability of the process. Therefore, the authors could not perform testing of antimicrobial activity since the results would not be relevant. However, the absence of synthetic fiber-forming polymer potential could shift the intended product toward highly advanced absorbable polymeric materials. According to the publications discussed above, where the antimicrobial activity of curcumin has already been experimentally proven, the same effect could be predicted for these samples as well.

Cellulose appears as a material in two forms, determined by their origin—bacterially produced nano-cellulose (spontaneously forming a fibrous structure) and cellulose isolated from woody plant parts (in powder form, requiring additional technological processing to be in fibrous form). Overall, cellulose is considered a suitable biopolymer for the development of advanced medical devices. A selection of cellulose and some other frequently used organic antimicrobial agents is summarized in Table 5. The most common application field of these materials is regenerative medicine, wound healing, or eventually, food packaging. 

### 3.2. Antimicrobial Biopolymers

A special case of natural antimicrobial agents are antimicrobial biopolymers. Antimicrobial biopolymers are attractive for the bioactivity of the native polymer, the ability to inhibit or kill the bacteria, and the biodegradability. The biodegradability makes polymers a more available material for customers in biomedical and industrial applications [103,104,105]. Among the biologically derived materials are one of the most common polymers obtained from nature—chitosan and a homopolymer, derived from a natural amino-acid, polylysine (PL). Both materials are extensively studied for their availability and inherent antimicrobial and antifungal activity [105].

#### 3.2.1. Chitosan

Chitosan has a good biocompatibility, biodegradability, high swelling, a low tendency to compromise the immune system, and good thermal stability [106]. The antimicrobial properties of CHIT consist of reactive amino groups attached to the main chain, whose positive charge interacts with the negatively charged bacterial surface, causing membrane disruption and bacterial breakdown [107]. It is the polycationic nature of the CHIT polymer solution that increases the surface tension of the polymeric solution, thereby increasing the electrical force required to overcome the repulsive electrostatic forces to form nanofibers from the solution. Fiber formation is also limited by the formation of hydrogen bonds due to the presence of many hydroxyl groups in the CHIT solution preventing the movement of the polymer chain during electrospinning [108,109]. Due to the presence of amino groups, chitosan is a weak base and insoluble at neutral pH. At acidic pH, chitosan becomes soluble, protonation of amino groups occurs, and it acquires a positive charge [110]. 

Pure CHIT solution can be electrospun using trifluoroacetic acid (TFA) as a solvent or dichloromethane (DCM) [111,112]. The use of TFA will result in the formation of salts with amino groups of chitosan, which will break the tight bond between individual chitosan molecules and thus facilitate electrospinning. The use of TFA is also advantageous because of its high volatility, rapid evaporation of the solvent, and rapid solidification of the resulting fibers [110]. However, due to the formation of these salts, the resulting chitosan nanofibers are rapidly soluble in neutral or basic solvents, which reduces their practical use. Therefore, cross-linking treatment is used to reduce the solubility of chitosan nanofibers [111,112]. Another solvent that can be used for the electrospinning of pure chitosan is acetic acid, where the use of the higher concentrations (90%, [113]) is advantageous in terms of reducing surface tension, viscosity, and electrostatic repulsion [109]. Pure CHIT nanofibers can also be obtained by processing the CHIT/synthetic polymer mixture, followed by removal of synthetic polymer. CHIT, in combination with PEO, is dissolved in the solvents mentioned in the previous paragraph and spun. After that is immersed in a liquid medium with base, neutralization occurs, and the membrane is stabilized and becomes insoluble in an aqueous environment. Then, the PEO is removed by immersion and washing the fibers in distilled water or in a water/ethanol mixture [114].

Another way to facilitate the electrospinning of CHIT is the use of auxiliary polymers because of the better polymer chain entanglement of CHIT and its increased flexibility. The most commonly used polymers include synthetic substances, such as PEO [114,115,116,117,118,119,120,121], PVA [12,110,122,123,124,125], PCL [74,126,127], copolyimide [128], or gelatin [129] (Table 6). The addition of these polymers improves the mechanical and physical properties of nanofibers [119].

Antimicrobial properties of formulations containing CHIT have been studied by various methods. Abid et al. (2019) [116] compared the antibacterial activity of chitosan nanofibrous matrix without and with the drug ciprofloxacin and zinc oxide (ZnO). The CHIT/PEO nanofibrous mat exhibited effects against both *S. aureus* and *E. coli*. The inhibitory zone of *E. coli* was larger than that of *S. aureus*, which can be explained by the thinner bacterial wall of the Gram-negative bacteria. The addition of ciprofloxacin resulted in a several-fold increase in antimicrobial efficacy while maintaining similar in vitro cytotoxicity. The publication also mentions the higher efficacy of the chitosan film compared to the nanofibrous form, which is explained by the higher amount of polymer incorporated into the film. The inhibition zone was also determined by Al-Musawi et al. (2020) [131], where the efficacy against bacterial strains of *Pasteurella multocida*, *Klebsiella rhinoscleromatis*, *Streptococcus pyogenes*, and *Vibro vulnificus* was compared against the antibiotics difloxacin and chloramphenicol.

The honey–chitosan nanofibrous matrix itself showed antimicrobial effects. With the addition of gold nanoparticles and capsaicin, the antimicrobial efficacy was increased several times. In a publication by Pandey et al. (2020) [124] dealing with chitosan nanofibers applied as a potential wound dressing or biodegradable packaging material, the efficacy against *E. coli* and *Listeria monocytogenes* was evaluated. After 24 h of incubation, efficacy was observed against both *E. coli* as well as against *L. monocytogenes*. Maximum efficacy was observed for the chitosan composition loaded with silver nanoparticles. The study of antimicrobial efficacy was also carried out in the publications of Anisiei et al. (2022) [114], Yu et al. (2021) [118], and Lungu et al. (2021) [121], where 6 mm to 10 mm nanofiber circles of CHIT/PEO were used as a control group, for which no antimicrobial activity was observed, unlike in previous publications. It can be assumed that the amount of chitosan used was not enough to create an inhibition zone. In the study provided by Ge et al. (2019) [117] were chitosan nanofibers loaded by other antimicrobial compounds. The CHIT nanofibrous matrix after 72 h showed enough antimicrobial activity against *S. aureus* and *E. coli*, in agreement with Abid et al. (2019) [116]. After the next 72 h, there was a rapid decrease in antimicrobial efficiency. In the study of Zhou et al. (2021) [127], CHIT nanofibers with PCL showed low antimicrobial efficiency against *E. coli* and *S. aureus*, in comparison with nanofibers loaded with rutin and quercetin.

The mentioned studies showed that for achieving maximum antimicrobial efficacy, it is not enough to rely only on the natural effects of CHIT polymer, even though it has effects at least in the short term. At longer time intervals, the cationic amino groups are saturated not only by the negatively charged surface of the bacterial walls [117] but also by the adsorption of non-specific proteins and undesirable biomolecules [132].

#### 3.2.2. Polylysine

Polylysine is a cationic homopolymer derived from the natural amino-acid lysine. It is a water-soluble, Food and Drug Administration (FDA) approved polymer, biodegradable, non-toxic, and resistant to high temperatures up to 120 °C. Polylysine has broad antibacterial and antifungal activity. The mechanism of action is based on the presence of cationic groups that are bound by electrostatic forces to the outer membrane of bacterial organisms with opposite charge, leading to their disruption. The polymer then enters the bacterial cell and induces the formation of reactive oxygen species, leading to DNA damage and subsequent cell death. The antibacterial activity increases with the number of repeating L-lysine residues [105,133]. Polylysine is plasticized in combination with natural or synthetic polymers, such as gelatin [134,135,136,137], PVP [138], PVA [133,139], PA [140], or PEO [141] (Table 7).

Lin et al. (2018) [134] tested the antimicrobial activity on a sample of beef meat inoculated with a culture of *L. monocytogenes*. The control group, in the form of gelatin nanofibers, showed no antimicrobial activity, in contrast to the nanofibers with polylysine and glycerin, which showed a reduction in the bacterial population at the storage temperature of 4 °C/10 days and a smaller reduction in colonization at 12 °C/7 days. The same research group prepared polylysine nanoparticles with cyclodextrin and thyme oil in gelatin nanofibers [135] and tested the antimicrobial activity against *Campylobacter jejuni* on a sample of chicken meat in the same way. Compared to polylysine and glycerin nanofibers in the previous study, a more significant inhibition of bacterial colonization was seen at 4 °C/7 days. They further tested the efficacy of nanofibers with polylysine and chitosan in the same way [141]. 

In a study conducted by Mayandi et al. (2020) [136], polylysine was electrospun with gelatin, followed by crosslinking via dopamine. In their experiments, they observed significant antimicrobial activity by determining the minimum inhibitory concentration (MIC). The antimicrobial activity against a wide range of bacterial strains was detected even after 35 days of soaking the nanofiber layer in PBS. The antibacterial activity was also monitored during in vivo testing on juvenile pigs, where induced burn wounds were inoculated with *P. aeruginosa* and *S. aureus*. For wounds covered with polylysine-containing nanofibers, detectable colonies were found in only 2 out of 5 and 1 out of 5 infected wounds, respectively, while wound closure was faster compared to the control. Amariei et al. (2018) [133] incorporated polylysine into nanofibers via the electrostatic adsorption method by immersing PAA-PVA nanofibers in polylysine-containing buffer. The antimicrobial activity was determined by MIC, the inhibition zone method, and by monitoring the colonization of the prepared wound dressings. Furthermore, the concentration during which a significant amount of oxygen radicals was generated was determined. The concentration was similar to MIC, and thus consistent with the role of ROS in polylysine toxicity. The antibacterial effect was evaluated to be the most effective against *Staphylococcus epidermidis* and the lowest against *E. coli*. The inhibitory zone was present in the first 24 h even around the control (pure PVA-PAA nanofibers), but after 14 days it was present only in the coating with polylysine, as shown by SEM images of nanofibers with and without polylysine. Similarly, the colonization of the wound dressings was more pronounced in the case of the dressings without polylysine.

Lan et al. (2021) [139] used the method of coaxial electrospinning for incorporating polylysine in the outer layer of nanofibers made of PCL for achieving an immediate antimicrobial effect and tea polyphenols in the inner layer of nanofibers made of PVA for a long-acting anti-ROS effect. The results of antimicrobial tests showed that only the nanofibers containing polylysine had an antibacterial effect. The nanofibers containing tea polyphenols had the opposite effect, which could not have the antibacterial effect due to the slow release from the inner core of the fibers. The matrix mimicking the structure of the PCL-PVA nanofibrous scaffold could promote the adhesion and proliferation of both bacterial strains *S. aureus* and *E. coli*. In contrast to the low cytotoxicity of tea polyphenols, polylysine showed almost no cytotoxicity due to the low charge of normal mammalian cells. In two published studies by Yang et al. (both in 2021) [99,142], a comparison of polylysine incorporated in starch nanofibers and hyaluronic acid nanofibers was conducted. Both formulations showed good biocompatibility and a broad antimicrobial spectrum. Due to the greater hydrophilicity of HA fibers, a greater amount of polylysine could be incorporated, and for the same reason, a faster release occurred compared to starch fibers. The HA fibers showed better mechanical properties, higher absorption, and faster degradation. Because of the bioactive effects, the formulation with HA was evaluated as more promising for the future applications in wound treatment.

The aforementioned studies indicate that polylysine could be a suitable source of a broad antimicrobial agent for wound management or even for food packaging, where it could be used as a preservative to reduce or slow down growth of the bacterial population or as an antimicrobial agent for wound healing, with promising results for positive cell viability.

#### 3.2.3. Antimicrobial Peptides

Antimicrobial peptides (AMP) represent an important role for multicellular organism as a key to defense against microbial proliferation and for modulation of the immune response. The main advantages of AMP include broad antibacterial activity, effectiveness against biofilm, little antibiotic resistance, rapid sterilization, and thermal stability. AMP are considered a safer alternative to silver-based wound healing products due to having natural amino acids as their degradation products and their short half-life. The antimicrobial effect is based on the adsorption of AMP on negatively charged bacterial membranes, followed by rupture and leakage of internal contents from the bacterial cell and subsequent apoptosis [132,143]. Specific studies dedicated to antimicrobial peptides are summarized in Table 8.

### 3.3. Synthetic Antimicrobial Agents

The third group of antimicrobials that can be incorporated into nanofibers are synthetic antimicrobial agents. Synthetic antimicrobial agents are chemical substances designed to inhibit or kill microorganisms, including bacteria, viruses, fungi, and protozoa. Their widespread application stems from their effectiveness, stability, and versatility in different formulations [148]. These substances, compared to natural-origin substances and inorganic nanoparticles, have the advantage of usually exhibiting stronger effects, thereby enhancing the overall efficacy of the final product. The most commonly used method of administering synthetic antimicrobials is either orally or in the form of a cream, or intravenous injection. Therefore, it typically involves systemic administration. Despite their utility, the use of synthetic antimicrobial ingredients raises several concerns and challenges that warrant careful consideration. One primary concern revolves around the potential development of antimicrobial resistance among microorganisms [149]. Prolonged exposure to synthetic antimicrobials can lead to the emergence of resistant strains, rendering these agents less effective over time. Moreover, there are growing environmental and health concerns associated with the use of synthetic antimicrobial ingredients, specifically antibiotics. These compounds can persist in the environment, accumulating in water and soil, where they may exert adverse effects on aquatic life and microbial organisms. Additionally, there is evidence suggesting potential health risks associated with prolonged exposure to certain synthetic antimicrobials. Furthermore, the efficacy of synthetic antimicrobial ingredients may vary depending on factors such as the formulation, concentration, and application method. Therefore, nanofibers are suggested as suitable carriers for these substances—they can be applied locally and might be comfortably and quickly changed by the medical staff. Simultaneously, the research trends bring about various new material combinations to prevent the development of antimicrobial resistance [5,150,151].

#### 3.3.1. Antibiotics

Antibiotics are a broad group of drugs that most often originate as a by-product of microorganism metabolism. Just like the previous group, they are very effective in fighting against bacterial infection. Since antibiotics have a very strict dosage plan during treatment, the easiest way to administer them is intravenously, directly into the bloodstream. However, antibiotics are mostly indicated per-orally, unless there are cases of acute systemic failure. Different types of antibiotics also have a different spectrum of action, regarding the microorganism against which they are used (broad-spectrum or targeted at specific strains).

By combining antibiotics with nanotechnologies—nanofibers—we obtain a very specific form that offers another way of applying these substances. The distribution of antibiotics in the nanofibrous network enables their local use into the healing/non-healing wound environment. At the same time, if the solution parameters are chosen well, the biomolecule is above all protected by a polymer matrix and, thus, the possible structural changes and activity limitations of the molecule due to the effect of high voltage are minimized. It has been proven that the kinetic profile of antibiotic release from the nanofibrous network was mostly gradual compared to the tablets, and their distribution to the site of action is more efficient in the long term, as the minimum effective concentration parameter is maintained throughout the therapy [152,153]. A selection of successfully incorporated antibiotics in nanofibrous carriers is summarized in Table 9. In general, the presented samples showed sufficient antimicrobial efficacy.

#### 3.3.2. Synthesized Antimicrobial Agents

The integration of synthesized antimicrobial agents into nanofibers has emerged as a cutting-edge approach in the field of wound healing. Nanofibrous materials provide a unique platform for the controlled release of synthetic antimicrobial compounds due to their high surface area-to-volume ratio and inherent biocompatibility. 

The synthetic antimicrobial agents exhibit potent activity against bacteria, fungi, and other microbes, thereby mitigating the risk of infections associated with open wounds. Additionally, in comparison with antimicrobial substances isolated from natural sources, synthetic antimicrobials exhibit the same or higher antimicrobial efficacy at lower concentrations. In practice, synthetic antimicrobial agents are frequently administered intravenously in the form of a solution or through local irrigation. However, the use of irrigation in wound therapy is not particularly advantageous, as the solution is mostly absorbed into the dressing material, endowed with absorbent properties, consequently diminishing the overall antimicrobial effect. On the other hand, the nanofiber structure promotes a conformal contact with the wound site, creating a protective barrier while concurrently fostering a stimulating environment for tissue regeneration. This innovative approach holds significant promise for advancing wound care, offering a multifaceted solution for infection control and accelerated healing. The synergy between synthesized antimicrobial agents and nanofiber matrices represents a pivotal stride toward the development of next-generation wound healing materials with improved therapeutic outcomes. A selection of synthesized and electrospun antimicrobials used worldwide is summarized in Table 10. 

Several research groups have focused on the combination of synthetic antimicrobial agents with nanofiber forms. Elsherbiny et al. (2022) [158] concentrated on preparing nanofibers based on cellulose acetate and lignin, incorporating various types of synthetic antiseptics—distinct copper-(II) complexes. The study revealed that not all combinations exhibited demonstrable antimicrobial efficacy. Among the evaluated samples, those containing the copper complex derived from the initial 2-Methoxy-4-(((4-(5,6,7,8-tetrahydronaphthalen-2-yl)thiazol-2-yl)imino)methyl)phenol and 2-Methoxy-4-((thiazol-2-ylimino)methyl)phenol were identified as the most effective for all bacterial types. Amoxicillin was used as a control. The efficacy was assessed against four strains—*P. aeruginosa*, *Acinetobacter baumannii*, *S. epidermidis*, and *Streptococcus faecalis*. In comparison, Anand et al. (2022b) [159] produced nanofibers using cellulose acetate and PCL, incorporating ferulic acid as the antiseptic. While the nanofibers showed satisfactory morphology, a uniformly homogeneous layer could not be obtained. Nevertheless, the authors prepared the samples exclusively for an in vivo study on diabetic rats. The nanofiber mats were tested against *S. aureus* and *P. aeruginosa* strains, demonstrating the composition’s efficacy against both bacterium types, with slightly higher efficacy against *S. aureus*. Bardoňová et al. (2021 and 2022) [150,151] focused on preparing nanofiber mats from modified hyaluronan designed for prolonged presence and structural stability at the healing site, while retaining the beneficial properties of HA. Antimicrobial agents, octenidine dihydrochloride and triclosan, were incorporated into the nanofibers. Material combinations were tested for varying proportions of the antimicrobial agent in the matrix, comparing their efficacy against *S. aureus*, *E. coli*, and *C. albicans*. For both triclosan and octenidine, the size of the inhibitory zone correlated with the concentration of the active substance, as evidenced in photographs of Petri dishes for the *S. aureus* strain. However, higher concentrations of antiseptics corresponded to significant cytotoxic effects on 3T3 fibroblasts. Consequently, the authors opted for lower concentrations of antiseptics in subsequent work. In the case of nanofiber layers with chitosan [160,162], the antimicrobial effect was increased, as demonstrated in previous texts with natural extracts and antibiotics. The effect was caused by the mere presence of this antiseptic biopolymer. 

## 4. Additional Remarks

Prevalent applications mentioned in the studied literature are in the field of medicine, such as wound healing and tissue engineering. This is probably caused by the focus on matrixes made of natural polymers, which often possess better biocompatibility and biodegradability than synthetic polymers. However, the electrospinning process might be more difficult to optimize and, thus, it is more favorable to prepare these nanofibrous materials for specialized applications than for applications requiring high throughput and low costs. The toxicity of some (mainly metallic) NPs or metal ions to human cells or their accumulation in human organisms is not described in detail; however, there is a small focus on studying the release of metal ions or leeching of NPs in the studied literature. The development of new materials’ research should go hand-in-hand with the studies of possible dangers, including the disposal of used materials with regard to the environment. Additionally, natural antimicrobiotics could possibly be dangerous if the dosage is not appropriate. In conclusion, the use of nanoparticles with antimicrobial activity, in combination with natural polymers, can be advantageous, especially in medicinal applications, where the use of antibiotics can be limited and thus struggle with bacteria antibiotic resistance. 

## Figures and Tables

**Table 5:** Summary of various organic antimicrobial compounds (with natural origin) incorporated in natural polymer-based nanofibrous mats.

Polymers	Additives	Incorporation	Antimicrobial Activity	Application	References
COL, CHIT	*Melissa officinalis* L. and *Anethum graveolens* L. essential oils	coaxial electrospinning	*S. aureus*, *E. coli*, *E. faecalis*, *S. typhimurium;* *C. albicans* *C. glabrata;* *A. brasiliensis*	wound dressing	Râpă et al. (2021) [91]
gelatin, PVA, PVP	*Ajwain* essential oil, aloe vera extract	coaxial spinning	*S. aureus*	wound dressing	Zare et al. (2021) [92]
gelatin, PVA	Propolis	electrospinning	*S. aureus*, *P. aeruginosa*	wound dressing	Ulag et al. (2021) [93]
sodium alginate, PVA, silk fibroin	asiaticoside	electrospinning	*S. aureus*, *P. aeruginosa*	wound dressing	Anand et al. (2022) [94]
sodium alginate	*Terminalia catappa* extract	electrospinning	human skin melanoma B16F10 cells	wound dressing	Muthulakshmi et al. (2022) [95]
cellulose acetate	*crude annatto* extract	electrospinning	in vivo testing on rats	wound healing	Antunes dos Santos et al. (2021) [96]
nanocellulose	lysozyme	spontaneous fiber formation	*S. aureus*	wound dressing	Silva et al. (2020) [97]
PU, carboxymethylcellulose	*Malva sylvestris* extract	electrospinning	*S. aureus*, *E. coli*	wound dressing	Almasian et al. (2020) [98]
sodium hyaluronate	ε-polylysine	solution freeze drying	*S. aureus*, *E. coli*	wound dressing	Yang et al. (2021) [99]
CHIT, PLGA	AuresinePlus (bacteriolytic enzyme)	electrospinning	*S. aureus*	wound dressing	Urbanek et al. (2021) [100]
CHIT, PCL	Jaft (internal layer of oak fruit)	electrospinning	*S. aureus*, *E. coli*	tissue engineering	Hashemi et al. (2021) [101]
gellan, PVA	eucalyptol/β-cyclodextrin inclusion complex	electrospinning	*C. albicans*, *C. glabrata*	wound healing	Mishra et al. (2021) [102]

**Table 6:** Nanofibers from chitosan blended with synthetic and natural polymers.

Polymers	Additives	Incorporation	Antimicrobial Activity	Application	References
PEO	-	Blend electrospinning	*E. coli*, *S. aureus*, *C. albicans*, *A. brasiliensis*	Wound healing	Anisiei et al. (2022) [114]
PEO	-	Blend electrospinning	-	-	Lemma et al. (2016) [115]
PEO	Ciprofloxacin, Zinc oxide	Blend electrospinning	*E. coli*, *S. aureus*	Burns, wound healing	Abid et al. (2019) [116]
PEO	Terpinen-4-ol	Blend electrospinning	*E. coli*, *S. aureus*	Long-term antimicrobial material	Ge et al. (2019) [117]
PEO	Antimicrobial peptide NP10	Blend electrospinning	*E. coli*, *S. aureus*	Wound healing	Yu et al. (2021) [118]
PEO	Vancomycin	Blend electrospinning	*S. aureus*, MRSA	Wound healing	Kalalinia et al. (2021) [119]
PEO	Bromelain	Blend electrospinning	-	Wound healing	Bayat et al. (2019) [120]
PEO	-	Blend electrospinning	*E. coli*, *S. aureus*, *C. albicans*, *C*. *glabrata*	Wound healing	Lungu et al. (2021) [121]
PVA	Zinc oxide	Blend electrospinning	*E. coli*, *P. aeruginosa*, *B. subtilis*, *S. aureus*	Wound healing	Ahmed et al. (2018) [12]
PVA	Honey, *Nepeta dschuparensis*	Blend electrospinning	-	Wound healing	Naeimi et al., (2020) [122]
PVA	*Echinacea purpurea* extract	Blend electrospinning	*S. aureus*	Wound healing	Fahimirad et al. (2023) [84]
PVP, PVA	Cu NPs	Blend electrospinning, co-electrospinning	*S. aureus*, *B. cereus*, *E. coli*, *P. aeruginosa*	Wound healing	Lemraski et al. (2021) [123]
PVA, PHB	-	Blend electrospinning	*E. coli*, *S. aureus*	Wound healing, drug carrier	Raza et al. (2023) [125]
PVA, PEO	-	Blend electrospinning	*E. coli*, *S. aureus*	Wound healing	Li et al. (2023) [130]
PCL	Dexpanthenol	Blend electrospinning	*P. aeruginosa*	Drug release	Wang et al. (2018) [74]
PCL	Tetracycline	Co-electrospinning	*E. coli*, *S. aureus*	Wound healing	Ghazalian et al. (2022) [126]
PCL	Rutin, Quercetin	Blend electrospinning	*E. coli*, *S. aureus*	Wound healing	Zhou et al. (2021) [127]
Copolyamide	-	Blend electrospinning	-	Wound healing	Shabunin et al. (2019) [128]
Gelatin	Graphene nanosheet	Blend electrospinning	*E. coli*, *S. aureus*	Wound healing	Ali et al. (2022) [129]
-	Tripolyphosphate, honey, gold nanoparticles, capsaicin	Blend electrospinning	*P. multocida*, *K. rhinoscleromatis*, *S. pyogenes*, *V. vulnificus*	Wound healing	Al-Musawi et al. (2020) [131]

**Table 7:** Electrospun nanofibers of polylysine blended with natural and synthetic polymers.

Polymers	Additives	Incorporation	Antimicrobial Activity	Application	References
Gelatin	Polydopamine	Blend	*S. aureus*, MRSA, *P. aeruginosa*, *E. coli*, *Acinetobacter*, *Enterococcus*	Wound healing	Mayandi et al. (2020) [136]
PVP, HA	Ferulic acid	Blend		Ocular Delivery	Grimaudo et al. (2020) [138]
Polyacrylamid, PVA		Electrostatic adsorption	*S. aureus*, *S. epidermidis*, *E. coli*	Wound healing	Amariei et al. (2018) [133]
PVA, PCL	Tea polyphenols	Coaxial electrospinning	*E. Coli*, *S. aureus*	Wound healing	Lan et al. (2021) [139]
Polyacrylamide	-	Blend		Improve to ES	Han et al. (2020) [140]
PEO, starch	-	Crosslinking	*E. coli*, *S. aureus*	Wound healing	Yang et al. (2021a) [142]
PEO, hyaluronic acid	-	Crosslinking	*E. coli*, *S. aureus*	Wound healing	Yang et al. (2021b) [99]

**Table 8:** Nanofibers containing antimicrobial peptides.

AMP	Polymers	Additives	Incorporation	Antimicrobial Activity	Application	References
Lysozym, Nisin	PAA, PVA	Glycerin	Electrostatic adsorption	*S. aureus*	Biomedical applications	Amariei et al. (2018) [132]
Tryptophan (Try)-rich peptide— Pac-525	Gelatin, CHIT	PLGA, HAp	Layer-by-layer electrospinning, electrospraying	*S. aureus*, *E. coli*	Coating materials of biomedical devices	He et al. (2018) [143]
CM11( cecropin melittin-derived)	CHIT, silk fibroin	-	Blend electrospinning	*E. coli*, *S. aureus*, *P. aeruginosa*	Wound healing	Khosravimelal et al. (2021) [144]
GH12-COOH-M2; AMP2	PEO	-	Centrifugal spinning	*S. epidermidis*	Biological dressing	Afshar et al. (2021) [145]
Pexiganan	PCL	-	Rinsing of PCL mats in PBS with pexiganan	*S. aureus*, *E. coli*	Wound healing	Chaiarwut et al. (2021) [146]
Cathelicidin peptide 17BIPHE2	PCL	-	Coaxial electrospinning	*S. aureus*, *K. pneumoniae*, *A. baumannii*	Sutures, coating materials of biomedical devices, or hemostasis materials	Su et al. (2019) [147]

**Table 9:** Summary of various antibiotic compounds incorporated in natural polymer-based nanofibrous mats.

Polymers	Additives	Incorporation	Antimicrobial Activity	Application	References
COL, PLA, PVP, PEO	cefazoline	coaxial spinning	*E. coli*, *S. aureus*, *P. aeruginosa.*	wound dressing	Hajikhani et al. (2021) [148]
gelatin, PVA	amoxicillin, peppermint oil	electrospinning	*E. coli*, *C. albicans*, *S. aureus*	wound dressing	He et al. (2022) [149]
poly(ω-pentadecalactone-*co*-ε-caprolactone), gelatin, CHIT	tetracycline hydrochloride	electrospinning	*S. aureus*, *B. subtilis*, *E. coli*	drug delivery	Turan et al. (2022) [154]
sodium alginate	halloysite nanotubes with cephalexine	electrospinning	*S. aureus*, *S. epidermidis*, *P. aeruginosa*, *E. coli*	tissue engineering	De Silva et al. (2018) [155]
cellulose acetate, PEO, silk fibrion	ciprofloxacin	electrospinning	*S. aureus*, *K. pneumoniae*, *P. aeruginosa*	wound dressing	Abdel Khalek et al. (2021) [156]
CHIT, PEO	vancomycin	electrospinning	*S. aureus*	wound healing	Kalalinia et al. (2021) [119]
CHIT, PCL	tetracycline hydrochloride	core–shell electrospinning	*S. aureus*, *E. coli*	wound healing	Ghazalian et al. (2022) [126]

**Table 10:** Summary of various organic antimicrobial agents incorporated in natural polymer-based nanofibrous mats.

Polymers	Additives	Incorporation	Antimicrobial Activity	Application	References
gelatin	poly([2-(methacryloyloxy)ethyl] trimethylammonium chloride)	electrospinning	*S. aureus*, *E. coli*, *methicillin-resistant S. aureus*, *A. baumannii*	wound dressing	Inal et al. (2019) [157]
cellulose acetate, lignin	N-vanillidene-phenylthiazole copper-(II) complex	electrospinning	*P. aeruginosa*, *A. baumannii*, *S. epidermidis*, *S. faecalis*	diaper dermatitis prevention	Elsherbiny et al. (2022) [158]
nanocellulose	lysozyme	spontaneous fiber formation	*S. aureus*	wound dressing	Silva et al. (2020) [97]
cellulose acetate, PCL, silk-sericin	ferulic acid	electrospinning	*S. aureus*, *P. aeruginosa*	wound dressing	Anand et al. (2022) [159]
hyaluronic acid derivative (lauroyl HA), PEO	octenidine dihydrochloride, triclosan	electrospinning	*S aureus*, *P. aeruginosa*	wound dressing	Bardoňová et al. (2021) [150]
hyaluronic acid derivative (lauroyl HA), native HA, PEO	octenidine dihydrochloride, triclosan	electrospinning	*S aureus*, *P. aeruginosa*	wound dressing	Bardoňová et al. (2022) [151]
CHIT, sodium alginate, PVA	deferoxamine	electrospinning	*S. aureus*, *P. aeruginosa*	wound healing	Jeckson et al. (2021) [160]
Quaternized CHIT, PVA	N-(2-hydroxy) propyl-3-trimethylammonium chitosan chloride	electrospinning	*E. coli*	wound healing	Wu et al. (2022) [161]
CHIT, cyclodextrin	triclosan	core–shell electrospinning	*S. aureus*, *E. coli*	wound healing	Ouerghemmi et al. (2022) [162]
silk fibroin, PEO, chondroitin sulfate	silver sulfadiazine	electrospinning	*E.coli*, *P. aeruginosa*, *B. subtilis*, *S. aureus*	wound healing	Cestari et al. (2022) [163]
shellac	monolaurin	electrospinning	*S. aureus*, *E. coli*	wound healing	Chinatangkul et al. (2018) [164]

## Data Availability

Not applicable.
